# Copy number variation of a gene cluster encoding endopolygalacturonase mediates flesh texture and stone adhesion in peach

**DOI:** 10.1093/jxb/erw021

**Published:** 2016-02-05

**Authors:** Chao Gu, Lu Wang, Wei Wang, Hui Zhou, Baiquan Ma, Hongyu Zheng, Ting Fang, Collins Ogutu, Sornkanok Vimolmangkang, Yuepeng Han

**Affiliations:** ^1^Key Laboratory of Plant Germplasm Enhancement and Specialty Agriculture, Sino-African Joint Research Center, Wuhan Botanical Garden of the Chinese Academy of Sciences, Wuhan 430074, China; ^2^Graduate University of Chinese Academy of Sciences, 19A Yuquanlu, Beijing 100049, China; ^3^Department of Pharmacognosy and Pharmaceutical Botany, Faculty of Pharmaceutical Sciences, Chulalongkorn University, Bangkok 10330, Thailand; ^4^College of Horticulture Science and Engineering, Shandong Agricultural University, Tai-An, Shandong 271018, China

**Keywords:** Copy number variation, flesh texture, melting flesh, peach, polygalacturonase, stone adhesion.

## Abstract

Texture is an important attribute affecting consumer perception of fruit quality. Peach melting flesh and flesh adhesion to stone (endocarp) are simply inherited and controlled by the *F-M* locus on linkage group (LG) 4. Here, we report that two genes encoding endopolygalacturonase (endoPG) in the *F-M* locus, designated *PpendoPGF* and *PpendoPGM*, are associated with the melting flesh and stone adhesion traits. *PpendoPGM* controls melting flesh while *PpendoPGF* has pleiotropic effects on both melting flesh and stone adhesion. The *F-M* locus has three allelic copy number variants of *endoPG*, H_1_ (*PpendoPGF* and *PpendoPGM*), H_2_ (*PpendoPGM*), and H_3_ (null). The H_2_ haplotype represents the ancestral one while the H_1_ and H_3_ haplotypes are two variants due to duplication and deletion of *PpendoPGM*, respectively. Accessions with H_1_H_1_, H_1_H_2_, or H_1_H_3_ genotypes show the freestone or semi-freestone and melting flesh phenotype, while both H_2_H_2_ and H_2_H_3_ accessions have the clingstone and melting flesh phenotype. The H_3_H_3_ accessions have the clingstone and non-melting flesh phenotype. Our study not only demonstrates a driving role of gene copy number variations in flesh texture diversification in fruit trees, but also provides a useful diagnostic tool for early seedling selection in peach breeding programmes.

## Highlight

Copy number variation at the *F-M* locus plays a driving role in flesh texture diversification in peach.

## Introduction

Texture is a sensory property that involves a variety of traits such as crispness, firmness, meltiness, and juiciness, therefore, it has an important direct influence on the consumer’s perception of fruit quality (Brookfield *et al.*, 2011). Major changes in fruit texture occur during ripening and are usually associated with softening. Fruit softening is primarily a result of the decline in cell wall strength and cell-to-cell adhesion. Numerous hydrolases have been suggested as being critical to cell wall disassembly in a variety of fruits such as tomato (Brummell and Harpster, 2001), strawberry (Quesada *et al.*, 2009), apple (Tacken *et al.*, 2010), and apricot (Leida *et al.*, 2011). However, increasing evidence showed that cell wall hydrolases related to fruit softening differ among species and their specific contribution to softening are still not clear (Prasanna *et al.*, 2007; Tomassen *et al.*, 2007).

Peaches are climacteric fruits and can be divided into melting flesh (MF) and non-melting flesh (NMF) types according to fruit softening behaviour. MF peaches lose flesh firmness gradually during early ripening and then soften rapidly (melting phase) in the late stages of ripening, whereas NMF peaches lack the melting phase and retain flesh firmness when fully ripe. Both MF and NMF peaches show considerable variation in firmness and texture although MF is completely dominant over NMF (Bailey and French, 1949; Monet, 1989). Based on flesh adhesion to the stone (endocarp), peaches are also classified as either freestone (F) or clingstone (C). However, the degree of adhesion can be varied as some peaches show semi-freestone or semi-clingstone (Bailey and French, 1949). Based on both flesh softening and stone adhesion, all peaches can be classified into three phenotypes, freestone melting flesh (FMF), clingstone melting flesh (CMF), and clingstone non-melting flesh (CNMF). The phenotype of freestone non-melting flesh (FNMF) has not been reported (Van Der Heyden *et al.*, 1997).

Both flesh texture (melting/non-melting) and stone adhesion (clingstone/freestone) in peach are simply inherited and controlled by the *Freestone (F*) and *Melting flesh (M*) loci, respectively. Several studies show that *M* and *F* are at the same locus which is designated *F-M* and mapped to a 3.5 cM interval on the bottom of linkage group (LG) 4 (Dettori *et al.*, 2001; Dirlewanger *et al.*, 2006; Ogundiwin *et al.*, 2009). A number of studies have been conducted to identify potential candidate genes for melting flesh and stone adhesion in peach. Initially, biochemical studies revealed that an endopolygalacturonase (endoPG) is highly expressed in ripe MF peaches, but extremely low in NMF peaches (Pressey and Avants, 1973; Lester *et al.*, 1996). Thus, the *endoPG* gene is deemed to be a candidate for the *M* locus in peach (Peace *et al.*, 2005; Morgutti *et al.*, 2006; Ghiani *et al.*, 2011). However, recombination between *M* and the *endoPG* gene was observed in three progeny derived from a cross between NMF and MF cultivars (Lester *et al.*, 1996). To reconcile this inconsistency, a hypothesis that the *F-M* locus may contain at least two copies of the *endoPG* gene was proposed (Peace *et al.*, 2007). One is responsible for melting flesh texture and another for stone adhesion. The clingstone and non-melting flesh phenotypes result from deletions in an *endoPG* gene cluster (Callahan *et al.*, 2004; Peace *et al.*, 2007). Overall, evidence for an endoPG cluster controlling melting flesh and stone adhesion in peach is strong but not conclusive.

Digital gene expression (DGE), a novel approach to profiling gene expression at the genome-wide level using next generation sequencing technology, has been widely used to identify genes for important horticultural traits in fruit trees such as citrus (Xu *et al.*, 2009), grape (Sweetman *et al.*, 2013), and strawberry (Kang *et al.*, 2013). The draft of the peach genome sequence was released in 2010 (Verde *et al.*, 2013) which provides an opportunity to perform DGE analysis. In this study, DGE profiling was conducted to analyse differential gene expression between different flesh phenotypes of peaches and candidate genes for the melting flesh and stone adhesion traits were validated using a candidate gene-based association strategy. Allelic variation of the *endoPG* gene cluster in the *F-M* locus was also investigated. Our goal is to clarify the genetic basis of stone adhesion and melting flesh in peach. Our study not only demonstrates that gene copy number variations play a driving role in phenotypic diversification in plants but also provides a simple diagnostic PCR test for assisted selection of stone adhesion and flesh softening in peach breeding programmes.

## Materials and methods

### Plant materials

All peach cultivars used in this study are maintained at the Wuhan Botanical Garden, Chinese Academy of Science (Wuhan, Hubei Province, China). Three peach cultivars, Nanshantiantao (FMF), Zhaohui (CMF), and Myojo (CNMF), were selected for DGE analysis. Fruit samples were collected at four developmental stages, fruitlet (S1), stone hardening (S2), pre-ripening (S3), and the ripening stage (S4), and the detail of each sample collection is listed in Supplementary Table S1 at *JXB* online. For each sample, ten fruits were collected, cut into small pieces, and mixed. Leaf samples were collected at a young stage in the spring season and used for genomic DNA extraction using the Universal Plant genomic DNA Extraction Kit (Tiangen, Beijing, China) according to the manufacturer’s instructions. Both fruit and leaf samples were immediately frozen in liquid nitrogen and stored at −75 °C until use.

### DGE library preparation and Illumina sequencing

DEG libraries were constructed from fruit samples of each cultivar at four different development stages, S1–S4. Total RNA was extracted using the Total RNA Rapid Extraction Kit (Zomanbio, Beijing, China) according to the manufacturer’s instructions. The purification of poly(A) mRNAs was performed using oligo-dT attached to magnetic beads. The purified mRNAs were fragmented using super sonication and then subjected to first- and second-strand cDNA synthesis using random hexamer primers. The DGE library was prepared using the Illumina gene expression sample preparation kit, and sequenced using the Illumina Hiseq2000 sequencer according to the manufacturer’s instructions.

### Sequence analysis and mapping of DGE reads

Sequencing-received raw image data was transformed by base calling into sequence data. These raw reads were stored in fastq format and then processed using in-house perl script. The frequency of error rate for RNA-Seq reads was calculated based on Phred score (Q_phred_). RNA-Seq reads were mapped to the peach reference genome v.1.0 (Verde *et al.*, 2013) and only 1bp mismatch was allowed. An index of the reference genome was built using Bowite V2.0.6 (Langmead *et al.*, 2009) and the mapping of RNA-Seq reads was performed using TopHat v 2.0.9 (Trapnell *et al.*, 2009).

### Differential gene expression analysis

Gene expression levels were calculated based on reads per kilobase per million mapped reads (RPKM). The number of clean reads mapped to each gene was counted using the software HTSeq v0.5.4p3. The read counts were standardized between samples by scaling the number of reads in a given library to a common value across all sequenced libraries using the edgeR program, version 2.6.10 (Robinson *et al.*, 2010). Differential expression analysis was performed using DEGSeq R package (1.12.0; TNLIST, Beijing, China). The *P* values were adjusted using the Benjamini and Hochberg method. A threshold *Q*-value of 0.005 and a log2-fold change of 1 was used to separate differentially expressed genes from non-differentially expressed genes. The sequences of the differentially expressed genes were compared against the NCBI RefSeq nucleotide database and the Swiss-Prot and UniPro protein databases. Differentially expressed genes were sequentially annotated according to the Blast results, followed by the pathway annotation pipelines, including GO (http://www.geneontology.org) and KEGG (www.genome.jp/kegg/).

### Quantitative real-time RT-PCR (qRT-PCR)

Total RNA was isolated using the Universal Plant Total RNA Extraction Kit (BioTeke, Beijing, China) according to the manufacturer’s instructions. The RNA samples were treated with DNase I (Takara, Dalian, China) to remove any contamination of genomic DNA. One microgram of total RNA per sample was subjected to cDNA synthesis using cDNA Synthesis SuperMix (TransGen, Beijing, China) according to the manufacturer’s instructions. A SYBR Green-based real-time PCR assay was carried out in a total volume of 25 μl reaction mixture containing 12.5 μl of 2× SYBR Green I Master Mix (Takara, Dalian, China), 0.2 μM of each primer, and 100ng of template cDNA. Melting curve analysis was performed at the end of 40 cycles to ensure proper amplification of the target fragments. Fluorescence readings were consecutively collected during the melting process from 60–90 °C at a heating rate of 0.5 °C s^−1^. Reaction mixtures without cDNA templates were also run as a negative control. All analyses were repeated three times using biological replicates. The difference in cycle threshold (Ct) between target and actin genes corresponded to the level of gene expression. A peach *GAPDH* gene was used as a constitutive control (Tong *et al.*, 2009), and all primer sequences are listed in Supplementary Table S2.

### Phylogenetic analysis of *endoPG* genes

Nucleotide sequences of *endoPG* genes in plants were used for phylogenetic analysis. DNA sequences were aligned using ClustalX and adjusted manually, as necessary. The resulting data were analysed using equally weighted Neighbor–Joining (NJ). The NJ trees were constructed using the heuristic search strategies of MEGA version 5. Bootstrap values were calculated from 1 000 replicate analyses.

### Thermal asymmetric interlaced PCR (TAIL-PCR) for unknown flanking sequences

Tail-PCR was performed according to a previously reported protocol (Liu and Chen, 2007). To recover the nucleotide sequences flanking the deletion of the *PpendoPGF* gene, three specific primers, designated DW-SP1, DW-SP2-adptor, and DW-SP3, were designed based on the sequences flanking the left side of the *F-box* gene in the *F-M* locus. Likewise, three specific primers, designated FBX-SP1, FBX-SP2-adptor, and FBX-SP3, were designed based on the coding sequence of the *F-box* gene to recover the nucleotide sequences flanking the deletion of the *PpendoPFM* and *PpendoPGF* genes. Tail-PCR was initially performed with DW-SP1 or FBX-SP1 and one of the four arbitrary degenerate primers containing an adaptor (LAD1-1 to LAD1-4) using genomic DNA as the template. The products from each first-round PCR were diluted 40-fold in double-distilled water (ddH_2_O) and then subjected to two sequential rounds of PCR amplification using two pairs of primers, DW-SP2-adptor or FBX-SP2-adptor/AC1 (an adaptor), and DW-SP3 or FBX-SP3/AC1, respectively. PCR amplification was conducted according to the same cycle parameters as reported by Liu and Chen (2007). All primer sequences are listed in Supplementary Table S3.

## Results

### Global gene expression patterns in different developmental stages of peach fruits

In this study, 12 DGE libraries were constructed and sequenced for fruit samples of three peach varieties, i.e. Nanshantiantao (NS), Zhaohui (ZH), and Myojo (MJ) at four developmental stages, i.e. fruitlet (S1), stone hardening (S2), pre-ripening (S3), and the ripening stage (S4). The raw reads were trimmed by removing adapter, empty reads, and low quality sequences. On average, approximately 8.8 million clean reads were generated for each library, with 0.44 Gb in size, and over 95% of the clean reads were mapped to the peach genome (Table 1). Reads mapped to unique or multiple locations of the reference genome were designated uni- or multi-reads, respectively. For each library, 91.3% and 4.5% of the clean reads were identified to be uni-reads and multi-reads, respectively.

**Table 1.** RNA-Seq reads and their physical mapping result in peach

Cultivar	Stage	No. of raw reads (Million)	No. of clean reads (Million)		Clean bases (Gb)	GC content (%)
			Single mapped	Multiple mapped		
NS	S1	10.61	9.51	0.67	0.53	45.62
	S2	8.26	7.53	0.31	0.41	45.82
	S3	8.76	8.06	0.26	0.44	46.22
	S4	8.36	7.80	0.23	0.42	46.03
ZH	S1	10.84	9.55	0.75	0.54	45.21
	S2	10.48	9.47	0.43	0.52	45.30
	S3	7.83	7.20	0.26	0.39	45.48
	S4	7.59	6.97	0.28	0.38	45.57
MJ	S1	8.18	7.18	0.60	0.41	45.24
	S2	7.66	6.94	0.35	0.38	46.55
	S3	9.39	8.65	0.30	0.47	45.60
	S4	7.95	7.33	0.29	0.4	45.77

**Fig. 1.**
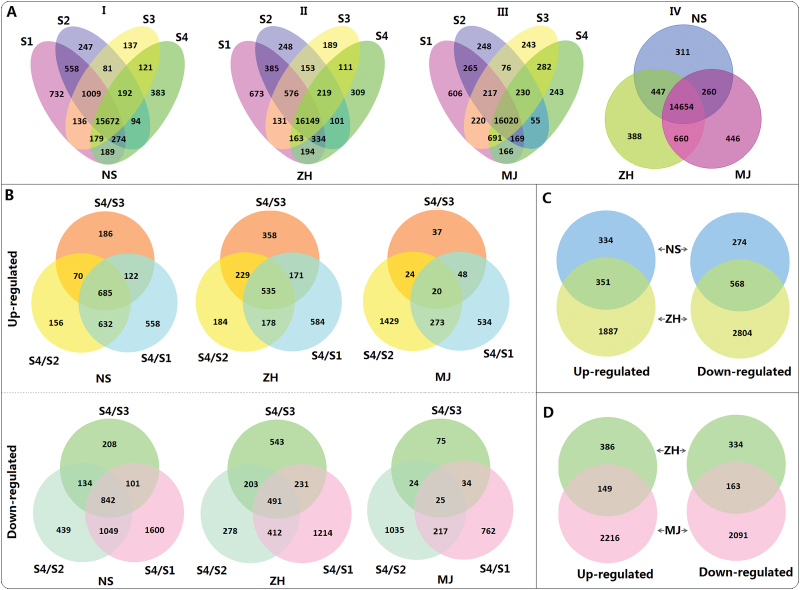
Identification of candidate genes responsible for stone adhesion and flesh melting in peach RNA-Seq based transcriptome analysis. (A) Venn diagrams showing the numbers of commonly and exclusively expressed genes in fruits throughout development (S1–S3) and ripening (S4) in cvs NS (I), ZH (II), and MJ (III), respectively, and the overlap between the commonly expressed genes in fruits of the three cultivars tested (IV). (B) Venn diagrams showing the numbers of genes commonly and exclusively up- or down-regulated in fruits of each cultivar tested between the developmental (S1–S-3) and ripening (S4) stages. (C) Comparison of up- and down-regulated genes in ripening fruits of clingstone cv. ZH with commonly up- and down-regulated genes, respectively, in ripening fruits of freestone cv. NS. (D) Comparison of up- and down-regulated genes in ripening fruits of non-melting cv. MJ with commonly up- and down-regulated genes, respectively, in ripening fruits of melting cv. ZH.

The clean reads were subjected to DGE analysis and the result revealed that 20 004, 19 908, and 19 731 genes were expressed in flesh tissues of cvs NS (FMF), ZH (CMF), and MJ (CNMF), respectively. For cv. NS, 18 749, 18 127, 17 527, and 17 104 genes were identified in flesh tissues of fruits at S1, S2, S3, and S4, respectively (Fig. 1A, I). A total of 15 672 genes were commonly expressed in all four stages, while 732, 247, 137, and 383 genes were exclusively expressed in S1, S2, S3, and S4, respectively. For cv. ZH, 18 605, 18 165, 17 691, and 17 580 genes were found in flesh tissues of fruits at S1, S2, S3, and S4, respectively (Fig. 1A, II). A total of 16 149 genes were commonly expressed in all four stages, while 673, 248, 189, and 309 genes were exclusively expressed at S1, S2, S3, and S4, respectively. For cv. MJ, 18 354, 17 280, 17 979, and 17 856 genes were identified in flesh tissues of fruits at S1, S2, S3, and S4, respectively (Fig. 1A, III). A total of 16 020 genes were commonly expressed in all four developmental stages, while 606, 248, 243, and 243 genes were exclusively expressed in S1, S2, S3, and S4, respectively. In addition, a total of 14 654 genes were commonly expressed in flesh tissues of fruits during the whole process of development in all three cultivars tested, whereas 311, 388, and 446 genes were exclusively expressed in flesh tissues of cvs NS, ZH, and MJ, respectively, during the whole process of fruit development (Fig. 1A, IV).

### Identification of candidate genes for stone adhesion and melting flesh in peach

Fruit stone adhesion and melting flesh occur in the ripening stage so DGE analysis was performed to identify genes differentially expressed between the fruit developmental (S1–3) and ripening (S4) stages (Fig. 1B). For cv. NS, 2 409 and 4 373 genes were up- and down-regulated, respectively, in flesh tissues of fruits during ripening. Of these genes, 685 and 842 were commonly up- and down-regulated in the S4 versus S1, S4 versus S2, and S4 versus*s* S3 comparisons, respectively. For cv. ZH, 2 238 and 3 372 genes were up- and down-regulated in flesh tissues of fruits during ripening, respectively. Among these genes, 535 and 491 were commonly up- and down-regulated, respectively, when compared between the ripening and developmental stages. For cv. MJ, 2 365 and 2 182 genes were up- and down-regulated in flesh tissues of fruits during ripening, respectively. Of these genes, 20 and 25 were commonly up- and down-regulated, respectively, when the ripening and developmental stages were compared.

Overall, 1 527 genes were commonly differentially expressed in flesh tissues of fruits of freestone cv. NS between the developmental and ripening stages, thus, these genes were expected to contain the *F* gene for freestone in peach. To narrow down the candidate gene list, the 1 527 differentially expressed genes were also compared with the differentially expressed genes between the developmental and ripening stages in clingstone fruits of cv. ZH. As a result, 334 up-regulated and 274 down-regulated genes were exclusively differentially expressed in the fruits of cv. NS (Fig. 1C; Supplementary Table S4). The *F* locus has been mapped to an interval flanked by two SSR markers EPPCU8503 and CPSCT005 on linkage group (LG) 4 (Dirlewanger *et al.*, 2006; Ogundiwin *et al.*, 2009). We checked the marker resources in the Genome Database for Rosaceae (GDR, http://www.rosaceae.org/search/markers) and found that the *F* interval is about 9.8Mb in physical size, ranging from 20.1Mb to 29.9Mb on LG4. Of the 334 up-regulated and 274 down-regulated genes, five (ppa000311m, ppa006857m, ppa025466m, ppa007187m, and ppa012362m) are located in the *F* locus.

**Fig. 2.**
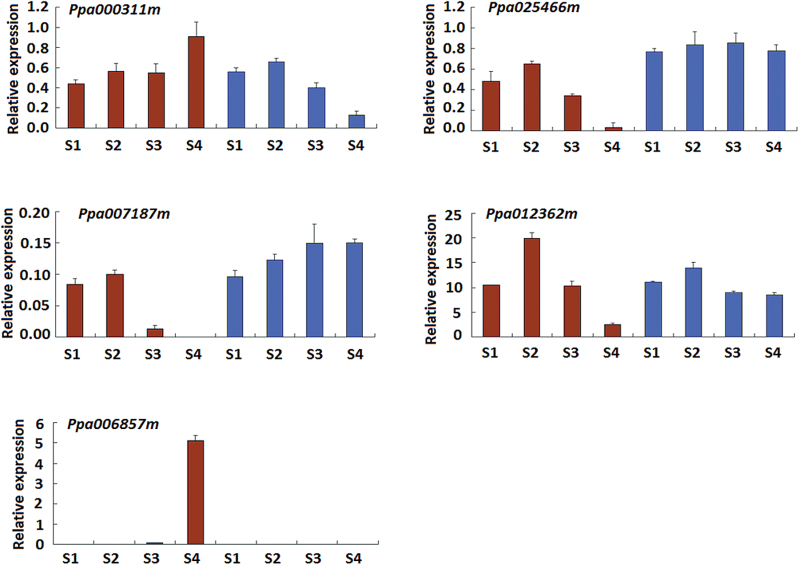
qRT-PCR validation of expression profiles of six genes in the *F* locus of peach. Red and blue colours represent cvs NS and ZH, respectively. S1 to S4 represent the four stages of fruit development.

The five genes in the *F* interval were subsequently subjected to qRT-PCR analysis. It is worth noting that one gene (ppa006857m) has a paralogue (ppa006839m, which will be described later) in the *F* locus. The two homologues are almost identical in coding sequences and they show only five single nucleotide polymorphisms (SNPs) in coding region (Supplementary Fig. S1). Three SNPs in the middle of the coding sequences were successfully used to develop qRT-PCR primers specific to ppa006857m and ppa006839m, and the primer specificity was validated by direct sequencing of qRT-PCR products. The qRT-PCR analysis showed that the expression profiles of all five candidate genes were consistent with the results of RNA-Seq based comparative transcriptome analysis (Fig. 2). However, only one gene (ppa006857m) encoding polygalacturonase was exclusively expressed in freestone fruits of cv. NS in the ripening stage and so it was deemed the candidate for stone adhesion.

**Fig. 3.**
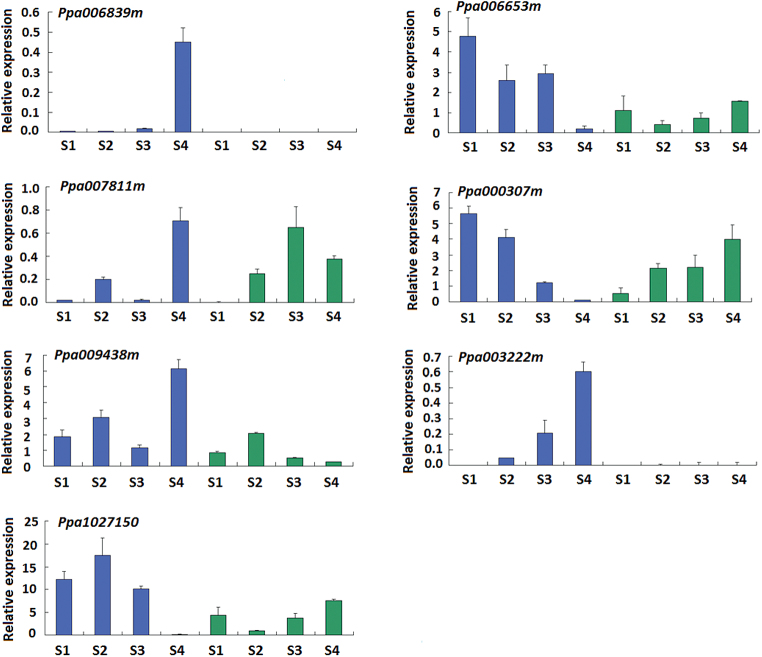
qRT-PCR validation of expression profiles of seven genes in the *M* locus of peach. Blue and green colours represent cvs ZH and MJ, respectively. S1 to S4 represent the four stages of fruit development.

Similarly, 1 026 genes were commonly differentially expressed in flesh tissues of fruits of melting flesh cv. ZH between the developmental and ripening stages and they were expected to contain the *M* gene for melting flesh in peach. The 1026 differentially expressed genes were also compared with the differentially expressed genes between the developmental and ripening stages in non-melting flesh fruits of cv. MJ and the result revealed that 386 up-regulated and 334 down-regulated genes were exclusively differentially expressed in the fruits of cv. ZH (Fig. 1D; Supplementary Table S5). Like the *F* locus, the *M* locus has also been mapped to the interval flanked by SSR markers EPPCU8503 and CPSCT005 on LG4 (Dirlewanger *et al.*, 2006; Ogundiwin *et al.*, 2009). Of the 386 up-regulated and 334 down-regulated genes, seven (ppa006653m, ppa000307m, ppa1027150m, ppa006839m, ppa007811m, ppa009438m, and ppa003222m) are located in the *M* locus. qRT-PCR analysis showed that the expression profiles of these seven genes in the *M* locus were consistent with the results of RNA-Seq based comparative transcriptome analysis (Fig. 3). However, only one gene (ppa006839m) was relatively highly expressed in ripening fruits of cv. ZH, but its transcripts were almost undetectable in fruits during development. Since the *ppa006839m* gene encodes polygalacturonase, it was considered as the candidate for melting flesh.

### Validation of the candidate genes for stone adhesion and melting flesh in peach

**Fig. 4.**
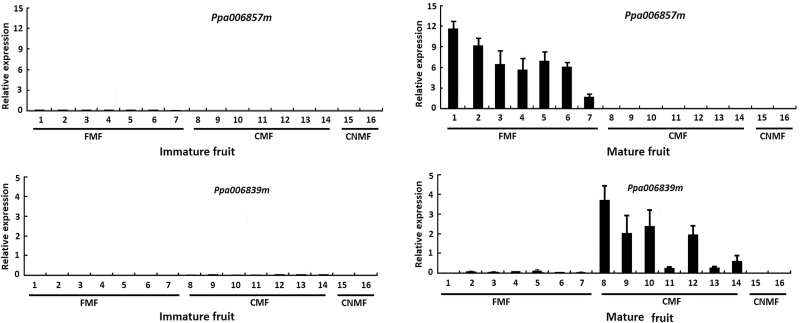
Expression profiling of two genes, *Ppa006857m* and *Ppa006839m*, in immature (S2) and ripening fruits of three types (FMF, CMF, and CNMF) of peach cultivars. The cultivars are as follows: 1, Early red 2; 2, Zhihebaitao; 3, F097NB; 4, Dalihehuangrou; 5, Ruiguangmeiyu; 6, Okitsu; 7, F725NB; 8, Huyou 002; 9, Hongfeng; 10, Jinxiang; 11, Reddomun; 12, Dubaifeng; 13, Hakuto; 14, Hujinmilu; 15, Xizhuan 1; 16, Long 124.

To determine whether *ppa006857m* and *ppa006839m* are strong candidates for stone adhesion and melting flesh, respectively, we conducted the following two experiments. Firstly, qRT-PCR analysis indicated that *ppa006857m* was exclusively expressed in ripening fruits of seven freestone cultivars, while its transcripts were almost undetectable in either ripening fruits of nine clingstone cultivars or immature fruits of all the 16 cultivars tested (Fig. 4). Similarly, the *ppa006839m* gene was expressed in ripening fruits of seven CMF cultivars, but its expression was extremely low to undetectable in either ripening fruits of FMF and CNMF cultivars or immature fruits of all the 16 cultivars tested (Fig. 4).

**Fig. 5.**
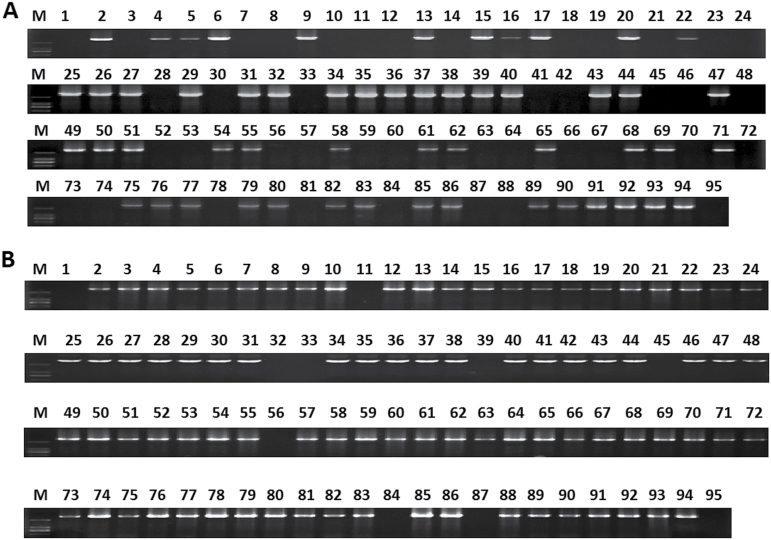
Agarose gel electrophoresis of the PCR products of the full-length genomic DNA of *Ppa006857m* (A) and *Ppa006839m* (B) genes in peach germplasm. The cultivars are indicated with the same numbers as listed in Table 2, and M indicates DNA ladders.

Secondly, two pairs of primers, P13 and P29 (Supplementary Table S6), flanking the whole genomic DNA sequences of *ppa006857m* and *ppa006839m*, respectively, were designed to amplify genomic DNA of the germplasm of 95 peach cultivars. A DNA fragment with an expected size of 2.9kb was present in freestone and semi-freestone cultivars but it was absent in clingstone cultivars (Fig. 5A). Similarly, a DNA fragment with an expected size of 3.0kb was detected in melting flesh cultivars but it was absent in non-melting cultivars (Fig. 5B). This demonstrated that the presence/absence of *ppa006857m* and *ppa006839m* is associated with stone adhesion and melting flesh, respectively.

**Fig. 6.**
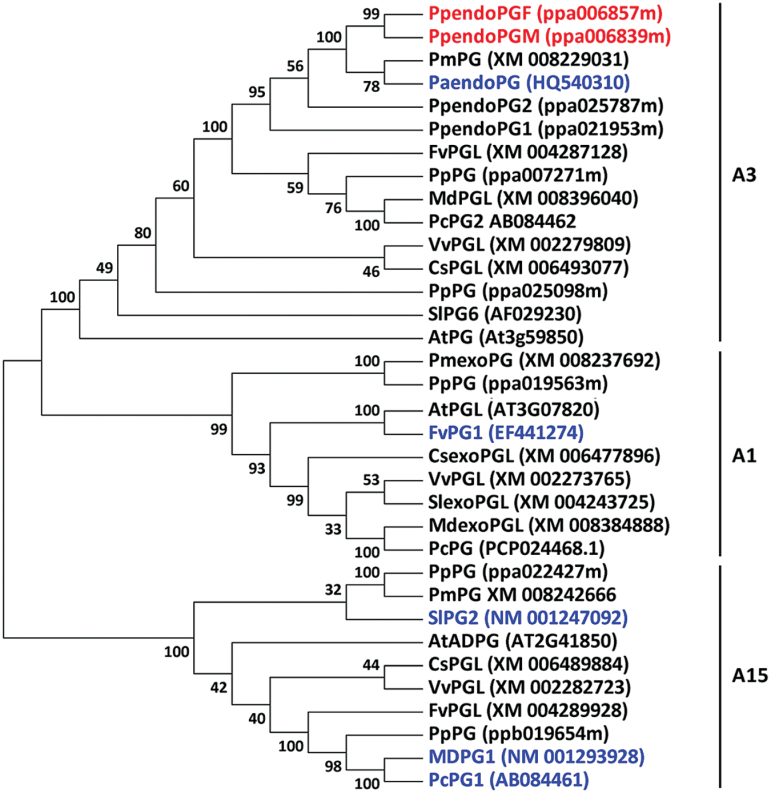
A phylogenetic tree derived from the nucleotide acid sequence of *PG* genes in both monocots and eudicots. Numbers near branches represent bootstrap values. The PG genes were named following the previously reported nomenclature system (Kim *et al.*, 2006). The GenBank accession numbers or the annotation accession numbers in the Phytozome v5.0 database (http:// www.phytozome.net) are indicated in brackets. The two *endoPG* genes indentified in this study are highlighted in red and the *PG* genes for fruit softening that have previously been reported in tomato, pear, strawberry, and apple are highlighted in blue.

Taken together, these results suggest that *ppa006857m* and *ppa006839m* are responsible for stone adhesion and melting flesh, respectively. In addition, phylogenetic analysis showed that *ppa006857m* and *ppa006839m* have diverged from previously reported *PG* genes for fruit softening in tomato (Chun and Huber, 1998) and Rosaceae species such as apple (Tacken *et al.*, 2010), pear (Hiwasa *et al.*, 2003), and strawberry (Quesada *et al.*, 2009), and they were designated *PpendoPGF* and *PpendoPGM*, respectively (Fig. 6).

**Table 2.** Characteristics of the stone adhesion and melting flesh traits in 95 peach cultivars

No.	Cultivar	Phenotype	No.	Cultivar	Phenotype	No.	Cultivar	Phenotype
1	Myojo	CNMF	33	Honghuatao	CNMF	65	Jinhualu	Semi-FMF
2	Nanshantiantao	FMF	34	R8A01	FMF	66	Zhaoxia	CMF
3	Zhaohui	CMF	35	SUNRAYCER	Semi-FMF	67	Beijing 2–7	CMF
4	Early red 2	FMF	36	754PS	Semi-FMF	68	F702NJ	Semi-FMF
5	NECTARED 4	FMF	37	Tiejing 1	FMF	69	F122NB	Semi-FMF
6	MARAVIHA	Semi-FMF	38	C243NB	Semi-FMF	70	Chuqiu	CMF
7	Jinxiu	CMF	39	Yixianbai	FMF	71	C227NS	FMF
8	Huaguang 2	CMF	40	Shengzhenbaitao	FMF	72	SUPPRISE	CMF
9	C209NB	Semi-FMF	41	Nagasawa Hakuho	CMF	73	Xinfeng	CMF
10	Huyou 002	CMF	42	Shimizu Hakuto	CMF	74	Hakuho	CMF
11	Xizhuan 1	CNMF	43	Dalihehuangrou	FMF	75	F098NB	FMF
12	FLORDAKING	CMF	44	Zaoyoutao	FMF	76	NECTARED 6	FMF
13	GREATDIAM	FMF	45	Dayebaitao	CNMF	77	VEGA	FMF
14	Hongfeng	CMF	46	Gailiangbaifeng	CMF	78	Hujinmilu	CMF
15	Zhihebaitao	FMF	47	F111PB	FMF	79	R6A09	FMF
16	H793NB	Semi-FMF	48	Reddomun	CMF	80	SUNRED	Semi-FMF
17	FLARDAGUARD	FMF	49	Tiejing 2	FMF	81	Yingshuang	CMF
18	Qianqu	CMF	50	F084PS	Semi-FMF	82	Tiejing 3	FMF
19	Jinxiang	CMF	51	Zaoyu	FMF	83	Okitsu	FMF
20	F718NB	Semi-FMF	52	Gangshanhong	CMF	84	Long 124	CNMF
21	R3PBPB	CMF	53	Dubaifeng	CMF	85	Jingyu	FMF
22	Yixianhong	FMF	54	Ruiguangmeiyu	FMF	86	F763NB	Semi-FMF
23	Xiahui 5	CMF	55	C226PB	FMF	87	Yanfeng	CNMF
24	Shanyibaitao	CMF	56	Zaohuangguan	CNMF	88	Qiubaimi	CMF
25	Dahongpao	FMF	57	Hakuto	CMF	89	NECTAROSS	FMF
26	Sunago wase	Semi-FMF	58	F127NB	Semi-FMF	90	FANTASIA	FMF
27	DISERFRED	FMF	59	Shenzhenhongmi	CMF	91	F084PS	FMF
28	Datuanmilu	CMF	60	Qiumi	CMF	92	F725NB	Semi-FMF
29	Shuho	Semi-FMF	61	F100NJ	Semi-FMF	93	Shinokubo	Semi-FMF
30	Ruiguang 27	CMF	62	Tuanchengzaosheng	Semi-FMF	94	F729NB	FMF
31	F097NB	FMF	63	Baihuashuimi	CMF	95	Jinyan	CNMF
32	C203NJ	FMF	64	Yingqing	CMF			

### Genomic structure variation of the *endoPG* gene cluster in the *F-M* locus in peach

The coding DNA sequences of *PpendoPGF/PpendoPGM* were blasted against the draft genome of peach cv. Lovell (Verde *et al.*, 2013), and the result revealed that *PpendoPGF/PpendoPGM* and their two homologues, termed *PpendoPG1* (ppa021953m) and *PpendoPG2* (ppa025787m), were located together within a 61kb region in the *F-M* locus (Supplementary Fig. S2). *PpendoPG1* and *PpendoPG2* showed 81.5% and 86.5% identities in coding DNA sequences with *PpendoPGF/PpendoPGM*, respectively. DGE analysis showed that both *PpendoPG1* and *PpendoPG2* were not expressed in fruits of the three tested cultivars, NS, ZH, and MJ, which suggests that *PpendoPG1* and *PpendoPG2* are not responsible for stone adhesion and flesh melting in peach.

**Fig. 7.**
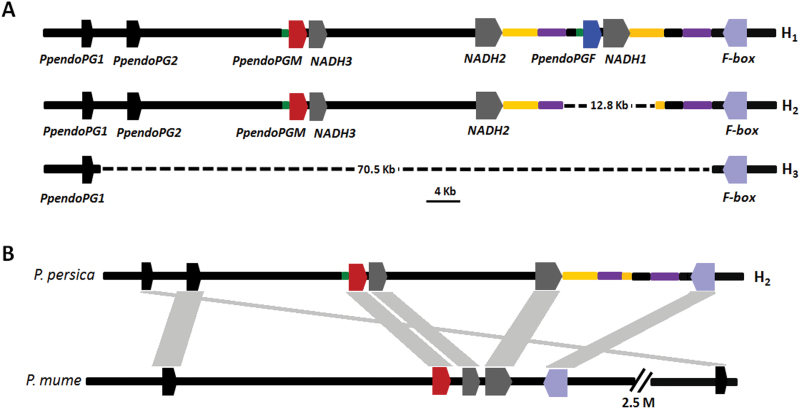
Genomic structure of the *F-M* locus in *Prunus*. (A) Three haplotypes in peach. The homologous regions are shown in the same colour. (B) Colinear genomic regions in *P. persica* and *P. mume*.

As mentioned above, both *PpendoPGF* and *PpendoPGM* show presence/absence variation. Thus, we investigated the genomic structure variation of the *endoPG* gene cluster in the *F-M* locus. Thirty-seven pairs of primers covering the *F-M* locus (Supplementary Fig. S2, Table S6) were designed based on the reference sequences of cv. Lovell and they were used to amplify cvs NS (FMF), ZH (CMF), and MJ (CNMF). For cv. NS, all these primers generated PCR fragments with the expected sizes. However, eight (P26–P33) and 27 (P2–P36) pairs of primers failed to generate any PCR products with the expected sizes using template DNA from cvs ZH and MJ, respectively. This indicates that a DNA fragment between primers P26 and P33 is lacking in cv. ZH, while a large-scale deletion is likely to occur between *PpendoPG1* and *F-box* genes in cv. MJ. Subsequently, PCR-based genome walking was conducted to identify the nucleotide sequence flanking each side of the deletion. As a result, a 12.8-kb gap covering *PpendoPGF* was detected in cv. ZH, and a large-scale gap of 70.5kb, which covers *PpendoPG2*, *PpendoPGF*, and *PpendoPGM*, was identified in cv. MJ (Fig. 7A). For ease of description, the allelic variants of the *endoPG* gene cluster in cvs NS, ZH, and MJ were designated H_1_, H_2_, and H_3_ haplotypes, respectively. The H_1_ haplotype consists of both *PpendoPGF* and *PpendoPGM*. The H_2_ haplotype contains *PpendoPGM*, but lacks *PpendoPGF*. The H_3_ haplotype lacks both *PpendoPGF* and *PpendoPGM*.

To facilitate genotyping the *F-M* locus, two pairs of primers, P38 and P39 (Supplementary Table S1), were designed to detect H_2_ and H_3_ variants, respectively. P38 and P39 were used to screen the germplasm of 95 peach cultivars (Supplementary Fig. S3). This result, together with those of the P13 and P29 primers as described above, revealed that all the accessions tested were grouped into three homozygous (H_1_H_1_, H_2_H_2_, and H_3_H_3_) and three heterozygous (H_1_H_2_, H_1_H_3_, and H_2_H_3_) genotypes. This suggests that four pairs of primers, i.e. P13, P29, P38, and P39, are enough for genotyping the *F-M* locus using a PCR-based diagnostic test. The majority of germplasm (55%) had the H_1_H_2_ genotype, while both H_1_H_1_ and H_1_H_3_ genotypes were detected in only one accession. The H_2_H_2_, H_2_H_3_, and H_3_H_3_ genotypes accounted for 21%, 14%, and 8% of all tested accessions, respectively. Moreover, the copy number of *PpendoPGF* and/or *PpendoPGM* in each accession was also quantified using qPCR-based copy number analysis (D’haene *et al.*, 2010), and the result (Supplementary Fig. S4) was consistent with that of the PCR-based genotyping method.

Moreover, we checked the genome of *Prunus mume* (Zhang *et al.*, 2012) and found a colinear genomic region to the peach *F-M* locus (Fig. 7B). The colinear genomic region in *P. mume* contains one copy of the *endoPG* gene, which is an orthologue of *PpendoPG*. Thus, the H_2_ haplotype in the *F-M* locus is likely to be an ancestral one, while the H_1_ and H_3_ haplotypes represent its two variants that result from duplication and deletion of the *PpendoPGM* gene, respectively.

In summary, the peach *F-M* locus consists of three allelic copy number variants, H_1_, H_2_, and H_3_. All accessions with H_1_H_1_, H_1_H_2_, or H_1_H_3_ genotypes show the freestone or semi-freestone and melting flesh phenotype, while both H_2_H_2_ and H_2_H_3_ accessions have the clingstone and melting flesh phenotype. The H_3_H_3_ accessions have the clingstone and non-melting flesh phenotype.

## Discussion

### Copy number variation of the *endoPG* gene in the *F-M* locus mediates the flesh texture and stone adhesion phenotypes in peach

Copy number variation (CNV) is defined as DNA segments ~1kb or larger that vary in copy number among haplotypes (Feuk *et al.*, 2006). CNV has been demonstrated to contribute substantially to the genetic diversity and to account for a significant proportion of phenotypic variation in humans (Sebat *et al.*, 2004; Stankiewicz and Lupski, 2010), animals (She *et al.*, 2008; Yalcin *et al.*, 2011), and plants (Cook *et al.*, 2012; Aliyu *et al.*, 2013; Iovene *et al.*, 2013; Maron *et al.*, 2013). Here, we provide an example of copy number variation of the *endoPG* gene in the *F-M* locus, which accounts for the diversification of flesh texture and stone adhesion in peach. The two-copy (H_1_), single-copy (H_2_), and null (H_3_) alleles of the *F-M* locus are present in FMF/semi-FMF, CMF, and CNMF cultivars, respectively, indicating a link between CNV and phenotype in peach. To our knowledge, our study represents the first report of important horticultural traits that are associated with gene copy number variations in fruit trees.

Of all the peach cultivars tested, 57%, 35%, and 8% carry two-copy, single-copy, and null alleles at the *F-M* locus, respectively. The low frequency of the null allele is mainly attributed to the fact that peach cultivars are mainly bred for fresh consumption. Freshly consumed fruits tend to be of melting flesh and, to a lesser extent, non-melting flesh (Peace *et al.*, 2007). The null allele conferring an undesired trait is likely to be eliminated by purifying selection in the breeding programme.This is consistent with a previous finding that CNV variants are under different levels of selection with deletions being under stronger purifying selection than duplications (Emerson *et al.*, 2008).

Screening peach germplasm reveals three alleles in the *F-M* locus. Interestingly, the single-copy allele consists exclusively of *PpendoPGM* which suggests that the *F-M* locus is a hot spot of mutation and the two-copy and null alleles are probably derived from duplication and deletion of *PpendoPGM*, respectively. *PpendoPGM* and *PpendoPGF* share 99% and 97% identity in coding and genomic DNA sequences, respectively, and the polymorphisms are mainly due to single nucleotide substitutions and small insertions and deletions. In other words, structural variation between *PpendoPGM* and *PpendoPGF* occurs at a similar frequency to that of allelic variation observed in the peach genome (Aranzana *et al.*, 2012). In addition, *PpendoPGM* and *PpendoPGF* share homologous sequences in both upstream and downstream regions. Thus, copy number variants in the *F-M* locus are likely to have arisen from replication slippage or retrotransposition (Hastings *et al.* 2009; Conrad *et al.*, 2010). The duplicated *endoPGM* gene has evolved into the *endoPGF* gene through synonymous and non-synonymous substitutions in coding sequences. Subsequently, chromosome recombination during self- and/or cross-fertilization results in six different genotypes in the *F-M* locus detected in this study.

The freestone and melting flesh phenotype is also present in other *Prunus* species such as apricot, plum, and sweet cherry (Peace *et al.*, 2007). Given the high level of synteny amongst the genome of *Prunus* species (Dirlewanger *et al.*, 2004; Jung *et al.*, 2009), it is possible that the flesh texture and stone adhesion phenotype in other *Prunus* species is also regulated by copy number variation of the *endoPG* gene.

### Functional divergence of two tandem-duplicated genes *PpendoPGF* and *PpendoPGM* in peach

As mentioned above, *PpendoPGF* is exclusively expressed in ripening fruits of freestone and semi-freestone cultivars and it co-segregates with the freestone and semi-freestone phenotype in all tested cultivars. This clearly suggests that *PpendoPGF* is responsible for stone adhesion in peach. It is worth noting that the expression level of *PpendoPGF* in ripening fruits of freestone cultivars is more than 5-fold higher than that of semi-freestone cultivars. Thus, the variation in degree of stone adhesion is probably related to the change of expression level of *PpendoPGF*, with a low expression level corresponding to the semi-freestone or semi-clingstone phenotype.

Similarly, *PpendoPGM* co-segregates with the melting flesh phenotype in all of the cultivars tested and its expression is associated with the melting flesh phenotype in clingstone cultivars. Moreover, temporal expression analysis shows that *PpendoPGM* is mainly expressed in the ripening stage which is in accordance with the fruit softening process. These results strongly suggest that *PpendoPGM* is a good candidate for melting flesh in peach. However, the transcripts of *PpendoPGM* are extremely low or undetectable in ripening fruits of melting flesh and freestone/semi-freestone cultivars where a high level expression is detected for *PpendoPGF*. Given the fact that freestone non-melting flesh has not been reported (Bailey and French, 1949; Van Der Heyden *et al.*, 1997), we speculate that *PpendoPGF* has a pleiotropic effect on flesh melting in peach. In summary, these findings suggest that the two tandemly duplicated genes *PpendoPGF* and *PpendoPGM* have diverged functionally. This is slightly different from a previous report that two *endoPG* genes in the *F-M* locus control melting flesh texture and stone adhesion, respectively (Peace *et al.*, 2007).

*PpendoPGF* and *PpendoPGM* show five nucleotide differences between their coding regions, of which only two at positions 146 (T or C) and 806 (A or G) result in amino acid substitutions at residues 19 (F or S) and 269 (T or S), respectively. These two amino acid substitutions lie outside the conserved region of endoPGs in plants (Supplementary Fig. S5), and are thus unlikely to be responsible for the functional divergence between *PpendoPGF* and *PpendoPGM*. Previous studies demonstrate that the diverse functions of *endoPG*s may be a consequence of differential expression (Torki *et al.*, 2000; Kim *et al.*, 2006). *PpendoPGF* shows a significantly higher level of expression in ripening fruits when compared with *PpendoPGM*. Therefore, it seems that the pleiotropic effects of *PpendoPGF* may be attributed to its high level of expression.

In plants, divergence in expression occurs frequently between tandemly duplicated *endoPG*s (Kim *et al.*, 2006). This expression divergence is also observed for tandemly duplicated *endoPG*s on peach LG4 in this study. Among the four *endoPG*s at the *F-M* locus, *PpendoPG1* and *PpendoPG2* are not expressed in fruits, while *PpendoPGF* and *PpendoPGM* are expressed in ripening fruits. Interestingly, the expression of *PpendoPGM* is extremely low or undetectable when it is clustered with *PpendoPGF*. As mentioned above, *PpendoPGF* has the functionality of *PpendoPGM*. Thus, it seems plausible to suppose that the high expression of *PpendoPGF* provides a negative feedback to inhibit the transcription of *PpendoPGM*. In addition, the transcription of *endoPG*s is known to be ethylene-dependent in peach (Hayama *et al.*, 2006; Ziliotto *et al.*, 2008). The promoter sequences are quite different between *PpendoPGF* and *PpendoPGM* (Supplementary Fig. S6). Therefore, it cannot be excluded that the ethylene-related transcription factors involved in the regulation of *endoPG* transcription prefer to bind to the promoter region of *PpendoPGF*, resulting in the divergence in expression between *PpendoPGF* and *PpendoPGM*.

**Fig. 8.**
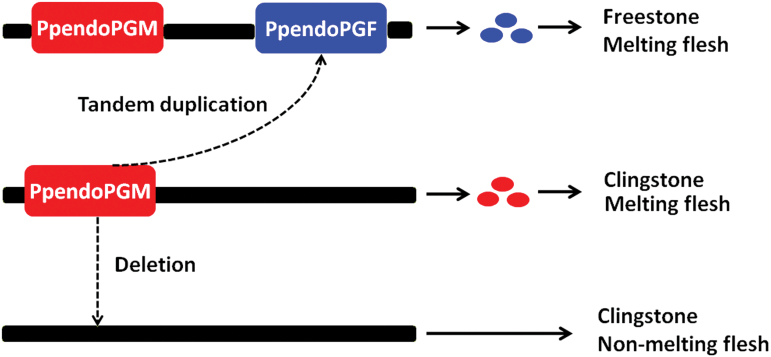
A proposed model underlying the diversification of flesh texture and stone adhesion in peach. The blue and red balls indicate PpendoPGF and PpendoPGM, respectively.

Taken together, we propose a model to account for stone adhesion and fruit softening in peach (Fig. 8). The diversification of the stone adhesion and melting flesh phenotype is mainly due to the presence/absence of two functional divergent *endoPG* genes, *PpendoPGF* and *PpendoPGM*, in the *F-M* locus of LG4 in peach. However, more studies are needed to identify the regulators controlling the transcription of *endoPG*s and the mechanism underlying the divergence in expression between *PpendoPGF* and *PpendoPGM* in peach.

### Multiple classes of genes encoding polygalacturonase involved in the regulation of fruit softening in Rosaceae fruit trees

Fruit softening is a result of modifications of cell wall polysaccharides’ architecture including the solubilization and depolymerization of pectin, the main cell wall component (Levy *et al.*, 2002; Brummell, 2006). During fruit softening, the activity of several enzymes increases significantly, including endoPG, endo-1,4-β-mannanase, α-l-arabinofuranosidase, and β-galactosidase (Brummell *et al.*, 2004; Hayama *et al.*, 2006). Of these enzymes, endoPG, a cell-wall degrading hydrolytic enzyme (Hatfield and Nevins, 1986), is well known to play an important role in fruit softening (Lester *et al.*, 1996; Callahan *et al.*, 2004; Peace *et al.*, 2005). In this study, we demonstrate that two *endoPG* genes are also responsible for flesh softening in peach.

Peach belongs to the Rosaceae family in which *PG* genes, such as *MdPG1* and *FaPG1*, have also been identified to be responsible for apple and strawberry fruit softening, respectively (Quesada *et al.*, 2009; Tacken *et al.*, 2010). Phylogenetic analysis indicates that the *PG* genes from peach, apple, and strawberry have diverged and belong to different clades. The plant *PG* genes are classified into three major groups (A, B, and C) and multiple clades (Kim *et al.*, 2006). *PpendoPGF*/*PpendoPGM*, *MdPG1*, and *FaPG1* are grouped into the same group A, but belong to A3, A15, and A1 clades, respectively. Apple, peach, and strawberry fruits represent different types of fruits which are pome, drupe, and berry fruits, respectively. Thus, the divergence of *PG* genes for fruit softening in various genus of the Rosaceae family is associated with fruit types. More studies are needed to clarify whether the mechanism underlying fruit softening has diverged amongst different types of fruit in Rosaceae.

## Supplementary data

Supplementary data can be found at *JXB* online.

Table S1. Fruit samples collected from three peach cultivars.

Table S2. Primers used for qRT-PCR analysis in peach.

Table S3. Primers used for DNA walking PCR in the *F-M* locus.

Table S4. Genes significantly differentially expressed between ripening stage (S4) and the three developmental stages (S1–S3) in fruits of two peach cultivars ‘NS’ and ‘ZH’.

Table S5. Genes significantly differentially expressed between ripening stage (S4) and the three developmental stages (S1–S3) in fruits of two peach cultivars ‘ZH’ and ‘MJ’.

Table S6. Primers used for screening allelic genomic variation at the *F-M* locus.

Fig. S1. Comparison of coding region sequences between the *Ppa006839m* and *Ppa006857m* genes. Fig. S2. Schematic diagrams of three types of *endoPG* gene clusters in the *F-M* locus on LG4 of peach.

Supplementary Fig. S3. Agarose gel electrophoresis shows the deletion of *PpendpPGF* (A) and *PpendoPGM* (B) in peach germplasm.

Fig. S4. Quantifying copy number of *PG* genes for melting flesh and/or stone adhesion in the *F-M* locus in peach using qRT-PCR.

Fig. S5. Alignment of the amino acid sequence of *PG* genes in plants. Fig. S6. Alignment of the promoter sequences of *PpendoPGM* and *PpendoPGF* genes in peach.

Supplementary Data
