# Supplementary material for: Copy number variation of a gene cluster encoding endopolygalacturonase mediates flesh texture and stone adhesion in peach
Source: J Exp Bot. 2016 Feb 5;67(6):1993–2005. doi: 10.1093/jxb/erw021 (PMC4783375; doi:10.1093/jxb/erw021)
Supplement: Supplementary Data [file supp_67_6_1993__index.html]

Copy number variation of a gene cluster encoding endopolygalacturonase mediates flesh texture and stone adhesion in peach — Copy number variation of a gene cluster encoding endopolygalacturonase mediates flesh texture and stone adhesion in peach — Supplementary Data 

# Copy number variation of a gene cluster encoding endopolygalacturonase mediates flesh texture and stone adhesion in peach

## Supplementary Data

Data files

- supplementary\_tables\_S1\_S3\_S6\_figures\_S1\_S6.pdf - Supplementary Data
- supplementary\_table\_S4.xls - Supplementary Data
- supplementary\_table\_S5.xls - Supplementary Data
