# Supplementary material for: Copy number variation of a gene cluster encoding endopolygalacturonase mediates flesh texture and stone adhesion in peach
Source: J Exp Bot. 2016 Feb 5;67(6):1993–2005. doi: 10.1093/jxb/erw021 (PMC4783375; doi:10.1093/jxb/erw021)
Supplement: Supplementary Data [file supp_erw021_supplementary_tables_S1_S3_S6_figures_S1_S6.pdf]

Copy number variation of a gene cluster encoding endopolygalacturonase mediates flesh texture and stone adhesion in peach

Chao Gu[1], Lu Wang[1], Wei Wang[1], Hui Zhou[1,2], Baiquan Ma[1,2], Hongyu Zheng[1,2], Ting Fang[1,2], Collins Ogutu[1,2], Sornkanok Vimolmangkang[1,3], Yuepeng Han[1,4*]

[1]Key Laboratory of Plant Germplasm Enhancement and Specialty Agriculture, Sino-African Joint Research Center, Wuhan Botanical Garden of the Chinese Academy of Sciences, Wuhan, 430074, China

[2]Graduate University of Chinese Academy of Sciences, 19A Yuquanlu, Beijing, 100049, China

[3]Department of Pharmacognosy and Pharmaceutical Botany, Faculty of Pharmaceutical Sciences, Chulalongkorn University, Bangkok 10330, Thailand

[4]College of Horticulture Science and Engineering, Shandong Agricultural University, Tai-An, Shandong 271018, China

*To whom correspondence should be addressed. E-mail: yphan@wbgcas.cn

Supplementary Table S1 Fruit samples collected from three peach cultivars

Stages	Cultivar		
	Nanshantiantao	Zhaohui	Myojo
Fruitlet (S1)	30 DAB	30 DAB	30 DAB
Stone hardening (S2)	52 DAB	54 DAB	55 DAB
Pre-ripening (S3)	65 DAB	82 DAB	79 DAB
The ripening stage (S4)	83 DAB	111 DAB	105 DAB

Supplementary Table S2 Primers used for qRT-PCR analysis in peach

Gene ID	Forward primer (5'→3')	Reverse primer (5'→3')
ppa000311m	CCAAGAGCCCGTGGTGTCAG	TCGCAGAGCAATGGGTAAGACT
ppa006857m	ATCCCTAAACAGCCAAATCTTCCAT	TGCCATCGGTGTTAGGGCTATTAC
ppa007187m	GTCGCATACCAAAAAGGACTCACTAC	CGGGTGCTGCCTCTCTTCCT
ppa012362m	AAGAGGCTGGCAACAAGTGG	CTTGTCCTTACCTTCGCATTCC
ppa025466m	AAATCCACAACCTTCCAACTCAG	GAGGGATGCCACAATCAACAG
Ppa006839m	TCCCTAATCAGCCAAATGTTCCAC	GCCATCGGTGTTAGGGCTGTTC
ppa003222m	TTCATGGGGCCTTTGTTGC	GGTCCATCCAGGTTTCATTCC
Ppa000307m	GCTGCTTCACCATCATTCGG	CAGGAGACTTAGAGGATTCCCAAC
ppa006653m	TGCTGGTTCCTCCATACATTGAC	GCTTAACAGGGTAGCCCACATC
ppa007811m	TTTGGGCAGTTATTGGCAGATT	GAGGAGTGGGTGGCTTTCGT
ppa009438m	GGCAGACAAGCACATTGGAAAG	CGGCACAGCACCCAATCATC
ppa1027150m	CATCAGAAATGTCAGGGCTTGG	AGGACTTGCTACTTCGTACCCACT
ppa006087m	CAATGCCATTCAAGCTAAGG	GAAATTCGATTTGCATGAGC

Supplementary Table S3 Primers used for DNA walking PCR in *F-M* locus

Name	Sequence	Note
DW-SP1	TCATTCGGGCTTTGTTGATTCTTG	Used in this study
DW-SP2-adptor	ACGATGGACTCCAGTCCGGCCATGGGACTTCGTTGCCCCTTTGTCTGAGC	Used in this study
DW-SP3	AGCCGAGTGAGGGAGTGTTGGTTG	Used in this study
FBX-SP1	AAGGCTTAGAGCACCACCAATCG	Used in this study
FBX-SP2-adptor	ACGATGGACTCCAGTCCGGCCATGGTTGGTGGTATGGTGTTGTGGGT	Used in this study
FBX-SP3	GAGGAGGGAAATGAGGCAGATGGG	Used in this study
LAD1-1	ACGATGGACTCCAGAGCGGCCGCVNVNNNGGAA	Liu and chen (2007)
LAD1-2	ACGATGGACTCCAGAGCGGCCGCBNBNNNGGTT	Liu and chen (2007)
LAD1-3	ACGATGGACTCCAGAGCGGCCGCVVNVNNNCCAA	Liu and chen (2007)
LAD1-4	ACGATGGACTCCAGAGCGGCCGCVDNBNNNCGGT	Liu and chen (2007)
AC1	ACGATGGACTCCAGAG	Liu and chen (2007)

Note: V= A/G/C; N= A/T/G/C; B=T/G/C; D= A/T/G.

Supplementary Table S6 Primers used for screening allelic genomic variation at the *F-M* locus

Name	Forward primer	Reverse primer	Size (kb)	Amplification region
P1	AACAATCGCATCGTCAATCTCC	AAGACCAGGCTCAGAGGAAACC	1.7	Whole sequence of *PpendoPG1*
P2	ACCTCACTTACAAGAATCAAGCAGC	GTCATGTGCGAAACTTAACTCTCG	2.1	0.1~2.3 kb; Downstream of *PpendoPG1*
P3	TGAGCACTCATGTACTAACCCAACC	TGTCCTTAGTACTGGAGGGCAAAC	2.3	Promoter sequence of *PpendoPG2*
P4	GGAAAAACCCGAAGTTGTGTGC	TGGGATTCACGATGATTCAGACTT	1.8	Whole sequence of *PpendoPG2*
P5	GATCAGATTGGAGAATGTGAAACTTAC	CATAGTTGACCTGCTGTGGTGTTT	2.3	0~2.2 kb; Downstream of *PpendoPG2*
P6	GGCAAGAAGTGGGATAACAAGG	GGTTGAGTAAAGGGGAGGGATTG	2.3	2~4.3 kb; Downstream of *PpendoPG2*
P7	CGCAAAGATCGTGAAGAAAGACT	CGGTTGGATCGTTGAATGGG	2.5	3.3~5.8 kb; Downstream of *PpendoPG2*
P8	CCCCATTCAACGATCCAACC	CACTGCTTTGCTTGATTGAGACC	2.3	5.7~8.0 kb; Downstream of *PpendoPG2*
P9	CACCAGTGGCTTGGCTTGAT	GTGCTACGACGGAGCCAATC	2.7	8.0~10.7 kb; Downstream of *PpendoPG2*
P10	ATGGGGCAATAGATGAATGAGC	ACTCGCAAGTGTACCAGGTCAAT	2.8	9.7~12.5 kb; Downstream of *PpendoPG2*
P11	GGCTGGAAAGATAAGACAATGACC	GGAATCACCTGGTTTGCCTTAG	2.9	11.4~14.3 kb; Downstream of *PpendoPG2*
P12	GCACATTCCCAAGCCAACC	CCTGCCATGTTAATCCTTACGTG	2.7	14.1~16.8 kb; Downstream of *PpendoPG2*
P13	ACTAACCCAACCAATTACTCAAACC	TGTCCTTAGTACTGGAGGGCAAAC	3.1	Whole sequence of *PpendoPGM*
P14	ACGAAGACATACACGGTACATCAGC	AGTCCAAAACTTTAGCCACTCCAT	2.3	0~2.1 kb; Downstream of *PpendoPGM*
P15	TCAGCAGCCAATCCACTTCC	TTGAACGCATCCAAGTCCTCTAC	3.4	1.6~5.0 kb; Downstream of *PpendoPGM*
P16	GGAAGGGACAAACATTTGAACATC	CACGGACACCACCAATCTCG	2.5	4.9~7.4 kb; Downstream of *PpendoPGM*
P17	GAGGAAGCCCAAAGACAGAGC	ACTGAGTAGTGACAGAAGGTCCCAT	3.4	6.1~9.5 kb; Downstream of *PpendoPGM*
P18	CAGTCCCCAAGTACACCAACCA	GCGAGACGGATGATTTATAGGTTC	3.3	8.3~11.6 kb; Downstream of *PpendoPGM*
P19	TGATGCGTGAGTATGTTGTGCG	CAAAGACCGCCTTCGACACG	3.4	10.6~14.0 kb; Downstream of *PpendoPGM*
P20	CGCCGCATTGGGAATCAGTA	CATGTAAAATGGGCAGTTTAGGGT	3.0	13.0~16.0 kb; Downstream of *PpendoPGM*
P21	ACAAATTGGAGTCGCAGGTTCT	GCCATAGCGATTTCAATAGCCT	3.1	14.5~17.6 kb; Downstream of *PpendoPGM*
P22	GTTCGACGGTGATGTCCTGC	TCCCCTTTTCAGTCTCACCTACC	3.9	16.3~20.2kb; Downstream of *PpendoPGM*
P23	TTCCACCCCGACCAACAAC	CCTAATGTAAGGCAGCATAAACTCC	3.6	18.9~22.5 kb; Downstream of *PpendoPGM*
P24	GTATAAAATGAGGTTAGGGAATGC	AGCAAGGGAGAAGGTTGTGGAC	2.3	22.0~24.3 kb; Downstream of *PpendoPGM*
P25	CCCTTCTTCCTTGTTTTCTTATGC	AGGAGCATTTCATACAACGCATC	3.1	24.2~27.3 kb; Downstream of *PpendoPGM*
P26	ATGCGTTGTATGAAATGCTCCTG	CGGGCTTTGTTGATTCTTGGAT	2.2	27.3~29.5 kb; Downstream of *PpendoPGM*
P27	TAACTCAGACAAAGGGGCAACG	ATCAGTGAAAACCTCCAACAAGC	1.8	29.4~31.6 kb; Downstream of *PpendoPGM*
P28	AGCACAATCTCTTTTCGCAATCAC	TGGAGTGGCTATGGCTGAGTTGAT	2.0	Promoter sequence of *PpendoPGF*
P29	TTTGAGGTTTGCTTCTTGTGATTAG	TGCCAACAACACAAGCTCATTC	3.3	Whole sequence of *PpendoPGF*
P30	ACGAAGACATACACGGTACATCAGC	CATATATTGGCTGCACGTTTACACC	1.4	0~1.2 kb; Downstream of *PpendoPGF*
P31	TCTTGGCTCTGTAATTGTAACGG	CTAATGTAAGGCAGCATAAACTCCAC	2.3	0.9~3.2 kb: Downstream of *PpendoPGF*
P32	AGGCAAGGAAAGATTGTTTATGTGG	GATAGAACCCTTCGGGCATTG	3.0	3.2~6.2 kb; Downstream of *PpendoPGF*
P33	TTGAATTACCACATCGACTTACAGAG	CCTTGTGGACCTCCGTAACCTTC	2.9	4.1~7.0 kb; Downstream of *PpendoPGF*
P34	AGGTTACGGAGGTCCACAAGG	GTTGGGTCCTTTACACTTGACTGAT	2.6	6.9~9.5 kb; Downstream of *PpendoPGF*
P35	TTCGTAGCAGCCCTCACCTC	TGGATGTGCTTTAGTGTTTGGGT	2.7	8.5~11.2 kb; Downstream of *PpendoPGF*
P36	AAGCCCCAACATAAATGACAAAACC	TTACGGGGGCATAAGGAAGC	3.0	11.2~14.2 kb; Downstream of *PpendoPGF*
P37	GGTCTCTTCTGCTGATGTTTGTGC	GCTACTTTACTTGTTCATTACTTGCCTC	2.8	14.2~17.0 kb; Downstream of *PpendoPGF*
P38	CAAAACCGAAACACTAAAGCACATC	AAGACCCACAAGTTTAGCAAATGAG		Used for *PpendoPGF* deletion
P39	AATCCCTGAAACAAGCAACCTG	TTCCTGAACTTAGACCCGATTTG		Used for *PpendoPGM* and *PPendoPGF* deletions
P40	AAGGCAAGCAAAGCCGTAGAT	GGTGGTGGGAAAGTTGAATGG		Used for qPCR (*PpendoPGM* and *PPendoPGF*)
P41	GGAGAAGTATGCCAATGACCAGG	AGTCACGCTTGTCCTCAGGGTAT		Used for qPCR (Inner control)

```
Ppa006839m  ATGGCGAACCGTAGAAGCCTCTTTTCTCTCTCACTTATCTTTGTGTTCATGATCAACTCAGCCATAGCCACTCCAGTCACATACAATGTGGCCAGTTTAG 100
Ppa006857m  ATGGCGAACCGTAGAAGCCTCTTTTCTCTCTCACTTATCTTTGTGTTCATGATCAACTCAGCCATAGCCACTCCAGTCACATACAATGTGGCCAGTTTAG 100

Ppa006839m  GAGCCAAAGCAGATGGCAAGACTGACTCCACAAAAGCCTTCCTCTTTGCATGGGCTAAAGCTTGTGCCTCGATGAATCCCGGTGTCATTTATGTGCCGGC 200
Ppa006857m  GAGCCAAAGCAGATGGCAAGACTGACTCCACAAAAGCCTTCCTCTCTGCATGGGCTAAAGCTTGTGCCTCGATGAATCCCGGTGTCATTTATGTGCCGGC 200

Ppa006839m  AGGAACGTTCTTTCTTCGCGATGTGGTGTTCAGTGGGCCTTGCAAGAACAATGCCATCACCTTCCGCATTGCCGGAACCCTTGTGGCCCCGTCGGATTAC 300
Ppa006857m  AGGAACGTTCTTTCTTCGCGATGTGGTGTTCAGTGGGCCTTGCAAGAACAATGCCATCACCTTCCGCATTGCCGGAACCCTTGTGGCCCCGTCGGATTAC 300

Ppa006839m  CGGGTCATCGGTAATGCAGCTAACTGGATTTTCTTTCACCATGTAAACGGGGTTACCATATCAGGTGGAATTCTTGACGGCCAAGGCACGGCCTTGTGGG 400
Ppa006857m  CGGGTCATCGGTAATGCAGCTAACTGGATTTTCTTTCACCATGTAAACGGGGTTACCATATCAGGTGGAATTCTTGACGGCCAAGGCACGGCCTTGTGGG 400

Ppa006839m  CTTGCAAGGCCTCTCATGGCGAGAGTTGTCCCAGCGGAGCAACGACTTTGGGTTTTTCCGACTCAAACAACATCGTGGTGAGTGGATTGGCATCCCTAAA 500
Ppa006857m  CTTGCAAGGCCTCTCATGGCGAGAGTTGTCCCAGCGGAGCAACGACTTTGGGTTTTTCCGACTCAAACAACATCGTGGTGAGTGGATTGGCATCCCTAAA 500

Ppa006839m  CAGCCAAATGTTCCACATAGTCATCAACGACTGCCAAAATGTGCAAATGCAAGGTGTCAGGGTTTCTGCTTCCGGGAACAGCCCTAACACCGATGGCATT 600
Ppa006857m  CAGCCAAATGTTCCATATAGTCATCAACGACTGCCAAAATGTGCAAATGCAAGGTGTCAGGGTTTCTGCTTCCGGTAATAGCCCTAACACCGATGGCATT 600

Ppa006839m  CATGTCCAAATGTCATCTGGTGTCACAATCCTCAACTCCAAGATTGCAACCGGTGACGATTGTGTCTCAATTGGCCCCGGAACCTCAAATTTGTGGATAG 700
Ppa006857m  CATGTCCAAATGTCATCTGGTGTCACAATCCTCAACTCCAAGATTGCAACCGGTGACGATTGTGTCTCAATTGGCCCCGGAACCTCAAATTTGTGGATAG 700

Ppa006839m  AAGGCGTTGCTTGTGGACCTGGCCATGGAATTAGCATTGGAAGTCTAGGCAAGGAGCAAGAAGAGGCCGGTGTACAAAATGTAACAGTTAAAACGGTTAC 800
Ppa006857m  AAGGCGTTGCTTGTGGACCTGGCCATGGAATTAGCATTGGAAGTCTAGGCAAGGAGCAAGAAGAGGCCGGTGTACAAAATGTAACAGTTAAAACGGTTAC 800

Ppa006839m  CTTTACTGGTACTCAGAATGGTCTAAGAATCAAGTCATGGGGGAGGCCAAGCACTGGGTTTGCTAGAAATATTCTTTTCCAACATGCTACAATGGTCAAT 900
Ppa006857m  CTTTAGTGGTACTCAGAATGGTCTAAGAATCAAGTCATGGGGGAGGCCAAGCACTGGGTTTGCTAGAAATATTCTTTTCCAACATGCTACAATGGTCAAT 900

Ppa006839m  GTCGAAAATCCTATTGTCATAGATCAACATTATTGCCCCGACAACAAAGGGTGCCCTGGTCAGGTTTCCGGAGTTCAAATTAGCGATGTGACATACGAAG 1000
Ppa006857m  GTCGAAAATCCTATTGTCATAGATCAACATTATTGCCCCGACAACAAAGGGTGCCCTGGTCAGGTTTCCGGAGTTCAAATTAGCGATGTGACATACGAAG 1000

Ppa006839m  ACATACACGGTACATCAGCAACAGAAGTTGCAGTAAAATTTGATTGCAGTCCCAAGCACCCTTGCAGCGAGATCAAATTGGAGGATGTGAAGCTTACTTA 1100
Ppa006857m  ACATACACGGTACATCAGCAACAGAAGTTGCAGTAAAATTTGATTGCAGTCCCAAGCACCCTTGCAGCGAGATCAAATTGGAGGATGTGAAGCTTACTTA 1100

Ppa006839m  CAAGAACCAAGCAGCTGAGTCTTCATGTAGCCATGCAGATGGAACAACTGAGGGTGTGGTTCAGCCTACAAGTTGTTTGTAG 1182
Ppa006857m  CAAGAACCAAGCAGCTGAGTCTTCATGTAGCCATGCAGATGGAACAACTGAGGGTGTGGTTCAGCCTACAAGTTGTTTGTAG 1182
```

Supplementary Fig. S1 Comparison of coding region sequences between the *Ppa006839m* and *Ppa006857m* genes. Single nucleotide polymorphism is highlighted in black background.

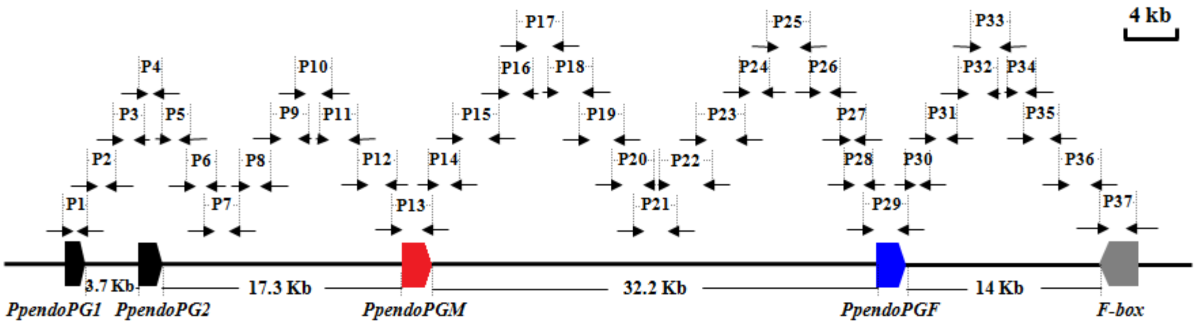

Supplementary Fig. S2 Schematic diagrams of three types of *endoPG* gene clusters in the *F-M* locus on LG4 of peach. P1 to P37 represent primers, whose corresponding PCR amplification fragments are indicated by two arrows.

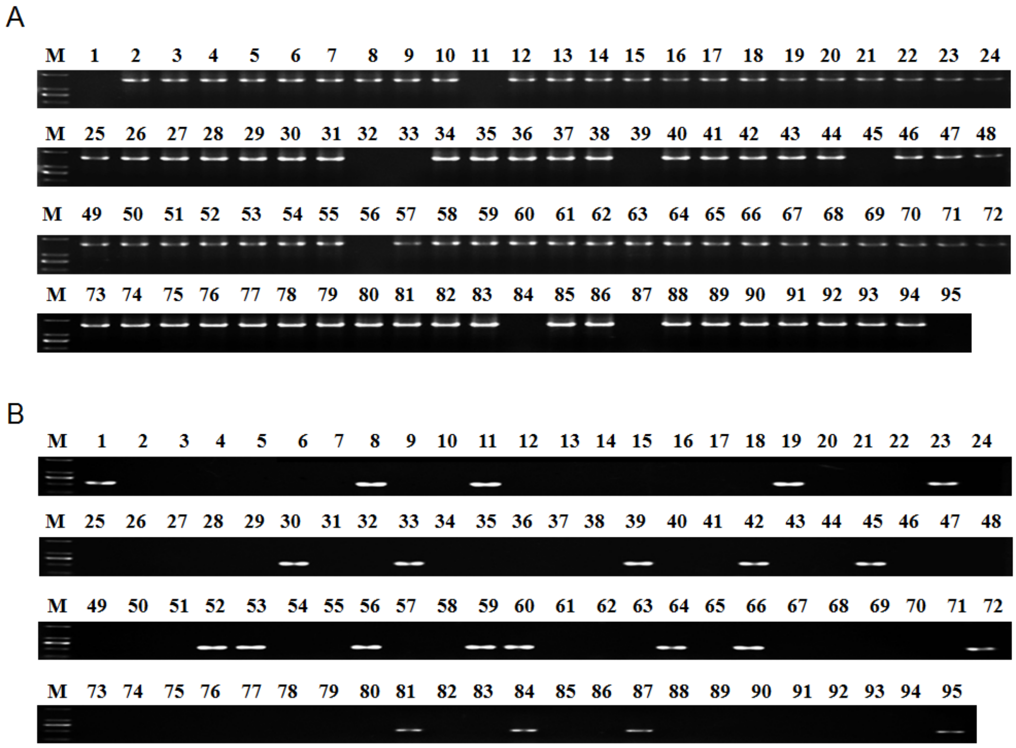

Supplementary Fig. S3 Agarose gel electrophoresis shows the deletion of *PpendpPGF* (A) and *PpendoPGM* (B) in peach germplasm. The cultivars are indicated with the same numbers as listed in Table 2.

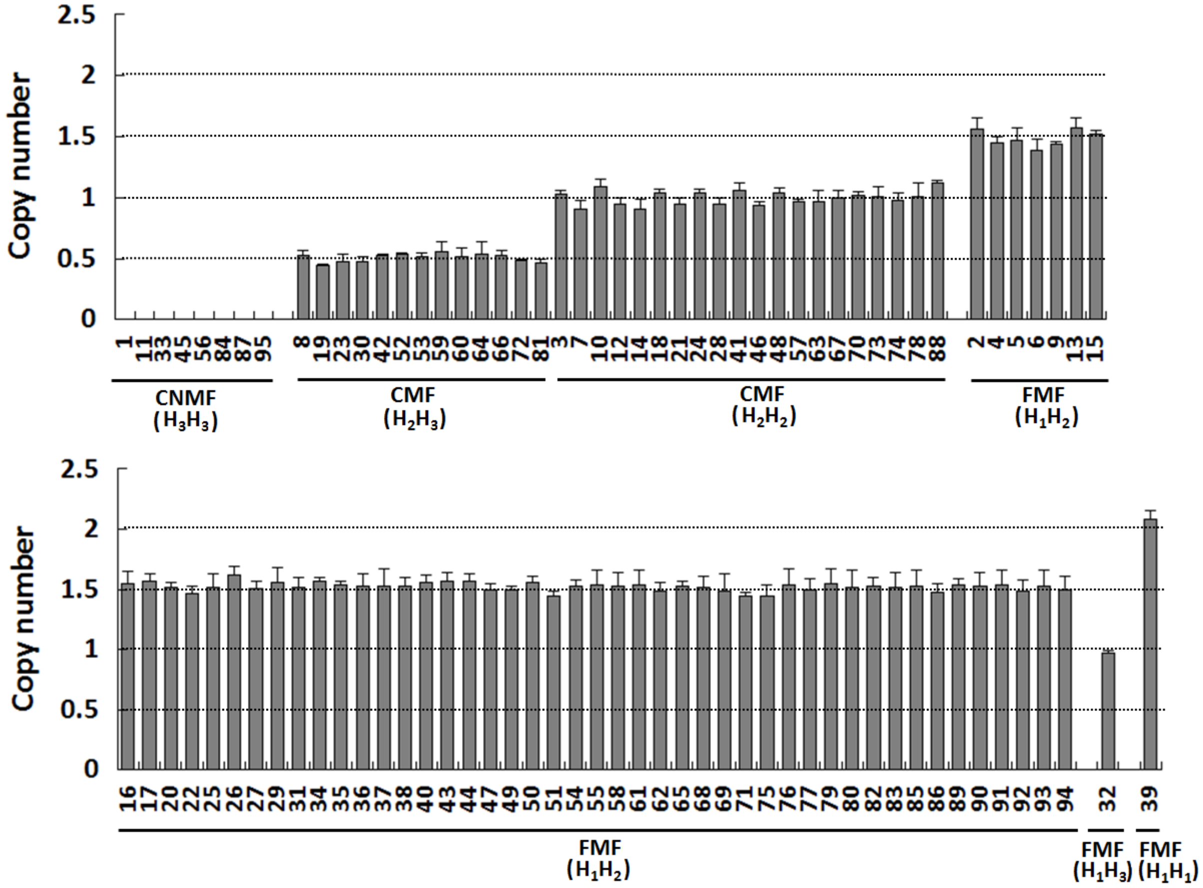

Supplementary Fig. S4 Quantifying copy number of *PG* genes for melting flesh and/or stone adhesion in the *F-M* locus in peach using qRT-PCR.

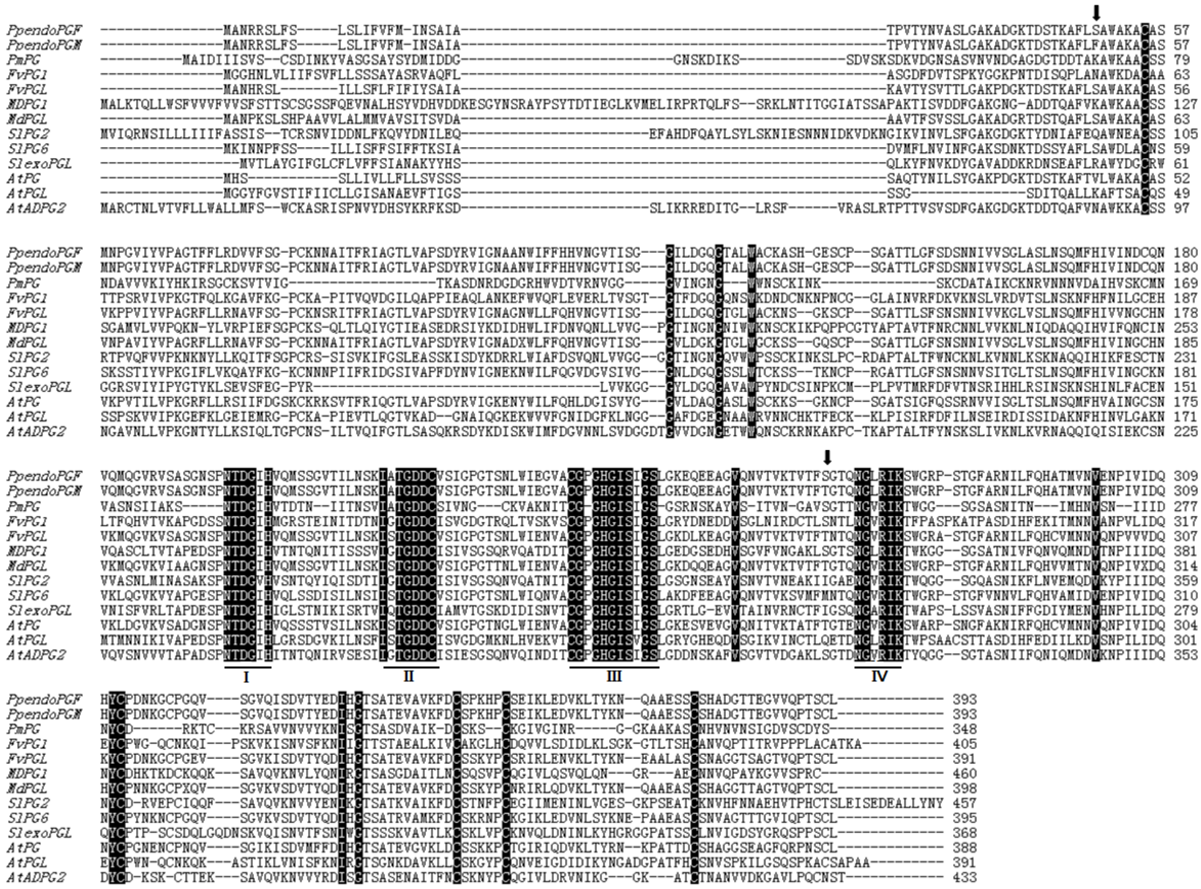

Supplementary Fig. S5 Alignment of the amino acid sequence of *PG* genes in plants. The conserved amino acid residues are highlighted in black background. I, II, III, and IV represent four conserved domains. The two different amino acid residues between PpendoPGF and PpendoPGM are indicated with black arrow.

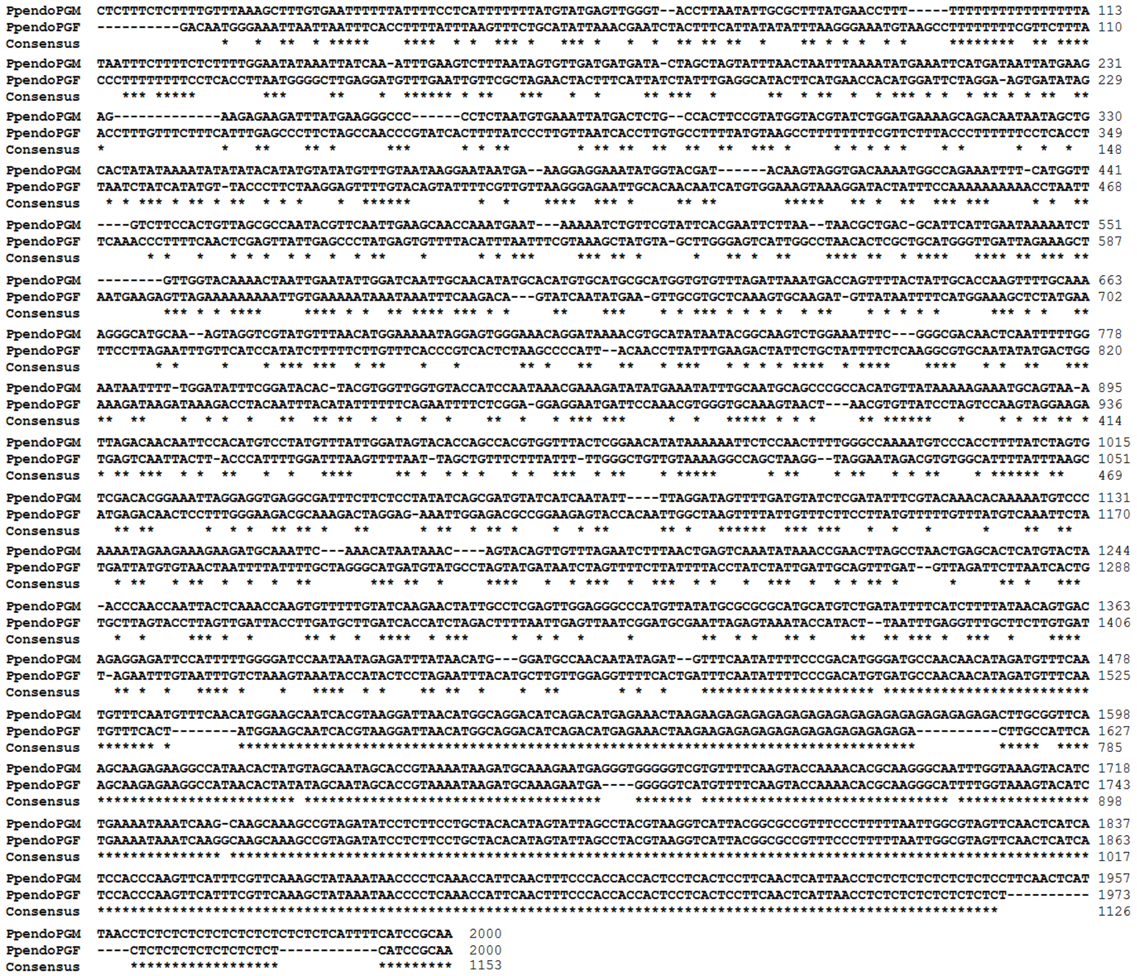

Supplementary Fig. S6 Alignment of the promoter sequences of *PpendoPGM* and *PpendoPGF* genes in peach.
